# Commentary: A Review of Prognosis Model Associated With Cardiogenic Shock After Acute Myocardial Infarction

**DOI:** 10.3389/fcvm.2022.856592

**Published:** 2022-02-23

**Authors:** Oriol Iborra-Egea, Cosme García-García, Antoni Bayés-Genís

**Affiliations:** ^1^ICREC Research Program, Health Sciences Research Institute Germans Trias i Pujol, Universitat Autònoma de Barcelona, Barcelona, Spain; ^2^Centro de Investigación Biomédica en Red Cardiovascular (CIBERCV), Instituto de Salud Carlos III, Madrid, Spain; ^3^Cardiology Service, Germans Trias i Pujol University Hospital, Badalona, Spain; ^4^Department of Medicine, Autonomous University of Barcelona, Barcelona, Spain

**Keywords:** cardiogenic shock, CS4P, biomarker, precision medicine, prognosis

## Introduction

Cardiogenic shock (CS) is a deadly complication of myocardial infarction (MI), as well as other cardiovascular diseases (CVD), including decompensated heart failure or myocarditis (1–3). CS is estimated to affect between 2 and 4 million people each year, and its incidence is growing rapidly, at a 9% compounded annual growth rate (4). CS mortality rates are close to 50%, and clinicians do not have efficient prognostic tools that help them manage these emergencies, leaving the patients (and their relatives) with no real information of their potential clinical evolution and burdening healthcare systems with poor capital allocation.

As for its management, CS requires admission to an intensive care setting and fluid resuscitation to correct hypovolemia and hypotension. Although it's very scarce, patients also need prompt initiation of pharmacologic therapy to maintain blood pressure and cardiac output (Aspirin/heparin, diuretics and Inotropic and/or vasopressor drug therapy) as well as early restoration of coronary blood flow (mainly via percutaneous coronary interventions) (5). Advanced therapies leveraging mechanical circulatory support devices (such as Veno-Arterial Extracorporeal Membrane Oxygenation [VA-ECMO], intra-aortic balloon pumps [IABP], Impella or TandemHeart) are being tested in clinical trials, and may pose the first life-saving solution for these patients (6), but these procedures are highly invasive and costly. Indeed, CS is estimated to average ~45.000 USD per patient of total hospital cost.

Early and accurate risk stratification is crucial for efficient identification of the sickest patients who may benefit from these advanced therapies. Unfortunately, no molecular tools for its prognostic have been developed yet. This hamper the physician's ability to optimize the patient's management, as well as restrict their clinical decision-making.

## The Importance of Reliable Prognostic Tools in Cs

There are several reasons why prognostic factors are important in CS. First, by determining which variables are prognostic of outcomes we gain insights on the biology and natural history of the disease. CS is not only determined by cardiac dysfunction, but a wide multisystemic failure instead, encompassing respiratory, hepatic, renal and inflammatory affectations. Taking this holistic view using unbiased biomarker discovery techniques, instead of the classic heart-centered approach, can lead to novel therapies for CS patients. Second, appropriate treatment strategies may be optimized and personalized based on the prognostic factors of an individual patient. Third, prognostic factors are often used in the design, conduct, and analysis of clinical trials to evaluate therapeutic advances as companion tests. Finally, patients and their families can be informed about the risk of recurrence or death and are empowered to take decisions that are more sensible.

## The CS4P Prognostic Tool

Currently, clinicians only have at their disposal clinical scores that compile classic biochemical, disease history and demographic variables to classify CS patients according to their mortality risk, and thus decide which therapeutic approach is better in each case. In an emergency situation, such as CS management, gathering these variables is impractical and time-consuming, which ultimately imply that this is implemented in very limited occasions. Moreover, many of these variables are difficult or impossible to obtain sometimes, and even when the medical team is able to acquire them, their predictive value is moderate and the variables themselves are static, meaning that cannot reflect the clinical evolution of the patient. For all these reasons, the decision-making process in CS is still mostly subjective to this date.

To aid this situation, our group used the latest advances in targeted proteomics and analyzed large, validated and independent cohorts, with the aim of finding molecular data that would be useful, simple to use and rapidly transferrable to the clinical setting (7–9). Here, we discovered that measuring a panel of just 4 proteins, we can confidently predict the outcome of a patient entering the emergency room suffering from CS (AUC 0.83). The CS4P score is based on the levels of liver fatty acid-binding protein, (L-FABP), beta-2-microglobulin (B2M), fructose-bisphosphate aldolase B (ALDOB), and SerpinG1 (IC1). These proteins are not cardiac-specific but reflect multi-organ dysfunction, systemic inflammation and immune activation. More importantly, the CS4P improved reclassification of 32% of patients when added to the CardShock risk score.

## Discussion

In this original review, Jingyue Wang et al. have made an impressive summary of the main prognostic strategies available to clinicians working on CS. The authors not only describe the two main clinical scores, and current gold-standard for CS prognosis, the IABP-Shock II score and the CardShock score, but also highlights broad severity scales, including APACHE, SAPS and SOFA scores, as well as general cardiovascular risk scores and CS-specific risk scores.

In this work, the CS4P stands out as the only fully molecular score, independent of any clinical variable, which bares several advantages over current alternatives. The proteins can be measured in a simple and rapid blood test without prior information of the patient, with much higher discrimination capabilities and can reflect the clinical evolution of the patient within hours, also assessing if the therapy under use is working in a personalized manner. However, the authors also state that, “due to the development of proteomics, its clinical application is limited.”

In both of our articles cited here, the authors failed to emphasize that we validated our proteomic results with Enzyme-Linked ImmunoSorbent Assay (ELISA) using external cohorts (CARDSHOCK patient cohort; *n* = 97), demonstrating its translation to the clinical setting. Albeit this was a retrospective validation, it demonstrates the potential of the CS4P to be used with gold-standard clinical practices.

As the authors rightly point out, there is still the need to perform prospective validations in order to understand the real prognostic value of the CS4P.

To this aim, our group is currently developing mice monoclonal antibodies (mAb) using complete recombinant proteins for each of our four target antibodies to immunize the mice. Four BALB/c strain mice have been immunized with the antigen mixed with adjuvant subcutaneously (*n* = 16 animals), with a minimum of 3 rounds of antigen administered. The animals were bled after the second and third immunization rounds and the determination of the titer was carried out by indirect ELISA. The two animals with the best titer were sacrificed and the B cells from the spleen were extracted for fusion with myeloma cells (obtaining the hybridomas). Said fusion was carried out by the classical method mediated by Polyethylene Glycol. General and differential screenings (to rule out cross reactivity) were also carried out by indirect ELISA.

We are developing these antibodies specifically to work in a ChemiLuminescence ImmunoAssay (CLIA) platform, which is much more sensitive and quicker than ELISA, and recently received public funding to validate this prototype. Such validation will start in 2022, and will include a comparison of our mAb vs. the ELISA tests from our original article to compare its performance. We will also investigate the values for the CS4P in retrospective cohort of healthy population (*n* = 100), as well as in post-MI patients classified by the Killip scale (*n* = 50 patients per Killip category).

Finally, we will use this data to determine an appropriate CS4P cut-off value for each category and start a pilot prospective study in CS patients (*n* = 40), which will allow us to monitor the patients at admission (*t* = 0), 6, 12, 24, and 48 h to reflect their clinical evolution during the first critical hours of CS.

## Author Contributions

OI-E has designed and written the article. CG-G has proof-read the article and contributed to the discussion. AB-G has supervised the manuscript, proof-read the article, and contributed to the discussion. All authors contributed to the article and approved the submitted version.

## Funding

This work was supported in part by grants from MICINN (PID2019-110137RB-I00, PLEC2021-008194), Instituto de Salud Carlos III (PIC18/00014, ICI19/00039, ICI20/00135, PI21/01700, PI21/01703), Red RICORS (PI21/01703), CIBERCV (CB16/11/00403) as a part of the Plan Nacional de I+D+I, and it was co-funded by ISCIII-Subdirección General de Evaluación y el Fondo Europeo de Desarrollo Regional (FEDER) and AGAUR (2017-SGR-483, 2019PROD00122).

## Conflict of Interest

OI-E, CG-G, and AB-G are co-inventors in a patent regarding the CS4P tool as a prognostic assay in cardiogenic shock (WO2020169751A1).

## Publisher's Note

All claims expressed in this article are solely those of the authors and do not necessarily represent those of their affiliated organizations, or those of the publisher, the editors and the reviewers. Any product that may be evaluated in this article, or claim that may be made by its manufacturer, is not guaranteed or endorsed by the publisher.
